# Medicinal Plants Used for Treating Reproductive Health Care Problems in Cameroon, Central Africa^1^

**DOI:** 10.1007/s12231-016-9344-0

**Published:** 2016-05-17

**Authors:** Roger Tsobou, Pierre Marie Mapongmetsem, Patrick Van Damme

**Affiliations:** Department of Plant Biology, University of Dschang, Dschang, Cameroon; Department of Biological Sciences, University of Ngaoundéré, Ngaoundéré, Cameroon; Department of Plant Production, Laboratory of Tropical and Subtropical Agriculture and Ethnobotany, Ghent University, Ghent, Belgium; Department of Crop Science and Agroforestry, Faculty of Tropical Agrisciences, Czech University of Life Sciences Prague, Prague, Czech Republic

**Keywords:** Ethnobotanical, medicinal plants, traditional healers, reproductive healthcare, venereal diseases, fertility problems, West Region Cameroon

## Abstract

**Medicinal Plants Used for Treating Reproductive Health Care Problems in Cameroon, Central Africa.** Approximately 80% of the African population uses traditional plants to deal with health problems, basically because of their easy accessibility and affordability. This study was carried out to document indigenous knowledge of medicinal plants used by traditional healers and elders in the treatment of reproductive health care in the Bamboutos Division of the West Region in Cameroon, Central Africa. The research methods used included semi–structured interviews and participative field observations. For the interviews, 70 knowledgeable respondents (40 traditional healers and 30 elders) were selected via purposive sampling. Voucher specimens were collected with the help of respondents, processed into the Cameroon National Herbarium in Yaoundé following standard methods, identified with the help of pertinent floras and taxonomic experts, and submitted to Department of Botany at the University of Dschang. Descriptive statistics were used to analyze and summarize ethnobotanical information obtained. Informant consensus factors (ICF) were used to elucidate the agreement among informants on the species to be used in the treatment within a category of illness. The results showed that a total of 70 plant species from 37 families (mostly of the Asteraceae [8 species], Euphorbiaceae [7], and Acanthaceae and Bignoniaceae [4 each]) are used in the treatment of 27 reproductive ailments, with the highest number of species (37) being used against venereal diseases, followed by female (29) and male infertility (21), respectively. Leaves (47.3%) were the most commonly harvested plant parts and the most common growth forms harvested were the herbs (45.7%), followed by shrubs (30%). Sixty percent of plant material was obtained from the wild ecosystems. Herbal remedies were mostly prepared in the form of decoction (66.2%) and were taken mainly orally. Informant consensus about usages of medicinal plants ranged from 0.5 to 1.0 with an average value of 0.91. It can be concluded that medicinal plants have played and will continue to play major roles in the management of reproductive healthcare in the study area.

**Electronic supplementary material:**

The online version of this article (doi:10.1007/s12231-016-9344-0) contains supplementary material, which is available to authorized users.

## Abstract (fr)

**Plantes médicinales utilisées dans le traitement des affections des organes reproducteurs au Cameroun, Afrique Centrale.** Approximativement, 80% de la population africaine utilisent les plantes pour résoudre leurs problèmes de santé. Cette large utilisation des plantes est attribuée à l’accessibilité et à leurs faibles coûts. Afin de contribuer à la documentation de ce riche héritage, une enquête ethnopharmacologique a été réalisée dans diverses localités du Département des Bamboutos, Ouest Cameroun, au sujet des plantes utilisées contre les affections des organes reproducteurs.Un total de 70 informateurs (40 guérisseurs et 30 personnes âgées)ont été consultés. A l’issu de l’enquête, 70 espèces de plantes appartenant à 64 genres et 37 familles botaniques avec une prédominance des familles des Asteraceae (8 espèces), des Euphorbiaceae (7), et des Acanthaceae et Bignoniaceae (4 espèces chacune) ont été répertoriées. Ces plantes sont employées pour traiter 27 affections des organes reproducteurs, avec en premier rang les maladies vénériennes qui utilisent 37 espèces, suivi de l’infertilité chez les femmes et les hommes, 29 et 21 espèces respectivement.La majorité (60%) de ces plantes est récoltée dans des jachères ou îlots forestières. Les types morphologiques les plus utilisés sont des herbes (45,7%), suivi des arbustes (30%). Les feuilles (47,3%) constituent la partie la plus utilisée, suivi des écorces (22,3%), plantes entières (19,7%), des tiges (5,2%), des tubercules (2,6%) et des inflorescences et fruits (1,3% chacun). La majorité de remèdes est préparée sous forme de décoction (66,2%) et sont majoritairement employées par voie orale, particulièrement en boisson. Les valeurs de consensus vis–à–vis des usages des différentes plantes sont comprises entre 0,5–1 avec une valeur moyenne de 0,91.Les plantes demeurent la seule source de médicament bio–disponible dans la zone d’étude et se verifie d’une part par l’usage accrue des herbes. Toutefois, elles peuvent servir de matière première pour la fabrication des médicaments modernes.

## Introduction

Cameroon is a lower middle–income country with a population of about 19.4 million, representing over 200 ethnic groups. Approximately 40% of its population lives below the one–dollar–per–day international poverty line. Life expectancy is 51 years (Cameroon Operational Plan Report or C.O.P.R. 2012). Cameroon’s epidemiological profile is dominated by communicable diseases such as malaria and HIV (prevalence of 5.5%), and an increasing prevalence of non–communicable afflictions, such as diabetes and cardiovascular diseases (C.O.P.R. 2012). The government of Cameroon currently allocates less than 6% of its national budget to health; this is far below the World Health Organization’s recommendation of 15% in order to meet the health sector’s Millennium Development Goals (MDGs). Multilateral and bilateral assistance has helped meet some of the nation’s health care needs and compensate for the public spending gap (C.O.P.R. 2012). Major infectious diseases are foodborne and waterborne; in addition, there are vector–borne diseases such as malaria. Access to modern medicine and doctors is low (1 per 13,000 inhabitants) (C.O.P.R. 2012).

In Cameroon, like in many other African countries, 80% of the population uses traditional medicine based on plants to improve its health state (Njamen et al. 2013). This reliance on medicinal plants can be explained partly by the high cost of allopathic drugs and inaccessibility of modern health institutions, but also by the cultural acceptability of the traditional system (Desissa and P. Binggeli 20000). However, as time goes on, traditional medicinal knowledge and its associated plants, which were developed for millennia, are subject to loss since they have been stored mainly in the memories of elderly people and handed down mostly by word of mouth over successive generations (Adjanohoun et al. 1996). Moreover, environmental degradation, deforestation, over–exploitation, over–grazing, agricultural land expansion, and acculturation continuously threaten Cameroon’s traditional medicinal plants and the associated knowledge (Simbo 2010). Hence, it is a timely endeavor to investigate, document, and analyze traditional knowledge of medicinal plants and the associated knowledge drivers so that sound medicinal plant utilization and management practices can be maintained. Furthermore, it provides the opportunity for recognition, promotion, management, and protection of indigenous medicinal plant knowledge of any community as a vital part of the nation’s heritage.

In this traditional system of medicine, plant preparations in the forms of decoctions, concoctions, macerations, or infusions are used to treat a wide range of diseases. Some of these plants are used in connection with human reproductive health problems, which are an important public health and social problem the world over (Diame 2010). In developing countries, particularly in Sub–Saharan Africa, the latter afflictions pose a major burden (Nordeng et al. 2013). Kamatenesi-Mugisha and Oryem-Origa (2007) have argued that reproductive health care is the second most prevalent health care problem in Africa.

In spite of the numerous publications on the country’s medicinal plants, including those by Focho et al. (2009) and Agbor and Naidoo (2011), attempts to document traditional uses of medicinal plants and their associated knowledge are insignificant when compared to the country’s 200 different ethnolinguistic groups, which have remained largely unexplored. The present research aims to fill at least part of this gap by documenting the wealth of indigenous knowledge related to the utilization of medicinal plants in the Bamboutos Division of the West Region in Cameroon, Central Africa for treating reproductive health care problems, a topic that has never been investigated before.

Traditional healers and elderly persons in the Bamboutos Division are expected to have rich knowledge of traditional medicine involving medicinal plants (Adjanohoun et al. 1996). Such knowledge is, however, under threat, just as is happening elsewhere in the country (Jiofack et al. 2010; Simbo 2010).

However, this study is vital in terms of gender equity to basic health care provisions and national development through indigenous knowledge innovations and bioprospecting. Further, plant species recorded will assist in the conservation of such plant species and may lead to the isolation of useful ingredients for the production of drugs and other medicinal consumables. Indeed, key plant species with high consensus on their use among respondents are reported to aid suitable plants for biological screening with expected higher success rate.

## Methods

### Study Area

**Fig. 1:**
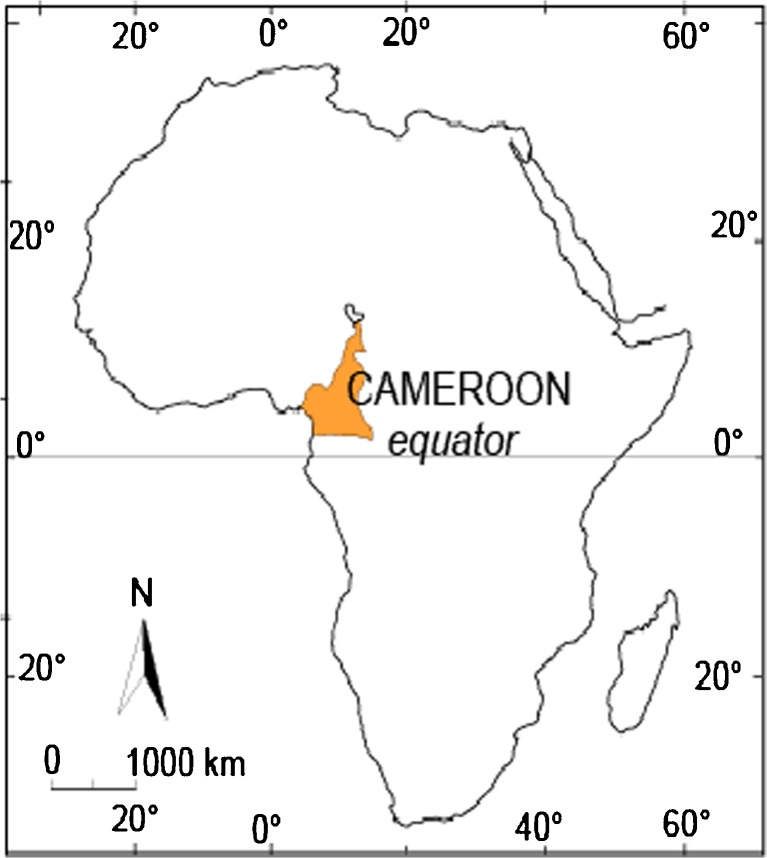
Africa map showing location of the study area: Cameroon, Central Africa.

**Fig. 2:**
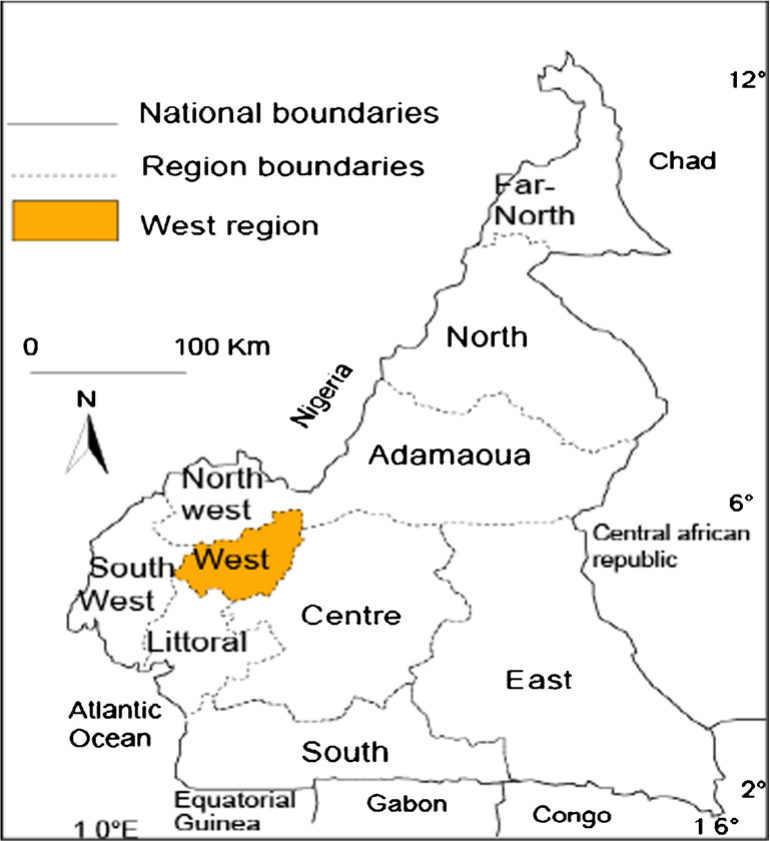
Map showing location of West Region within Cameroon.

**Fig. 3:**
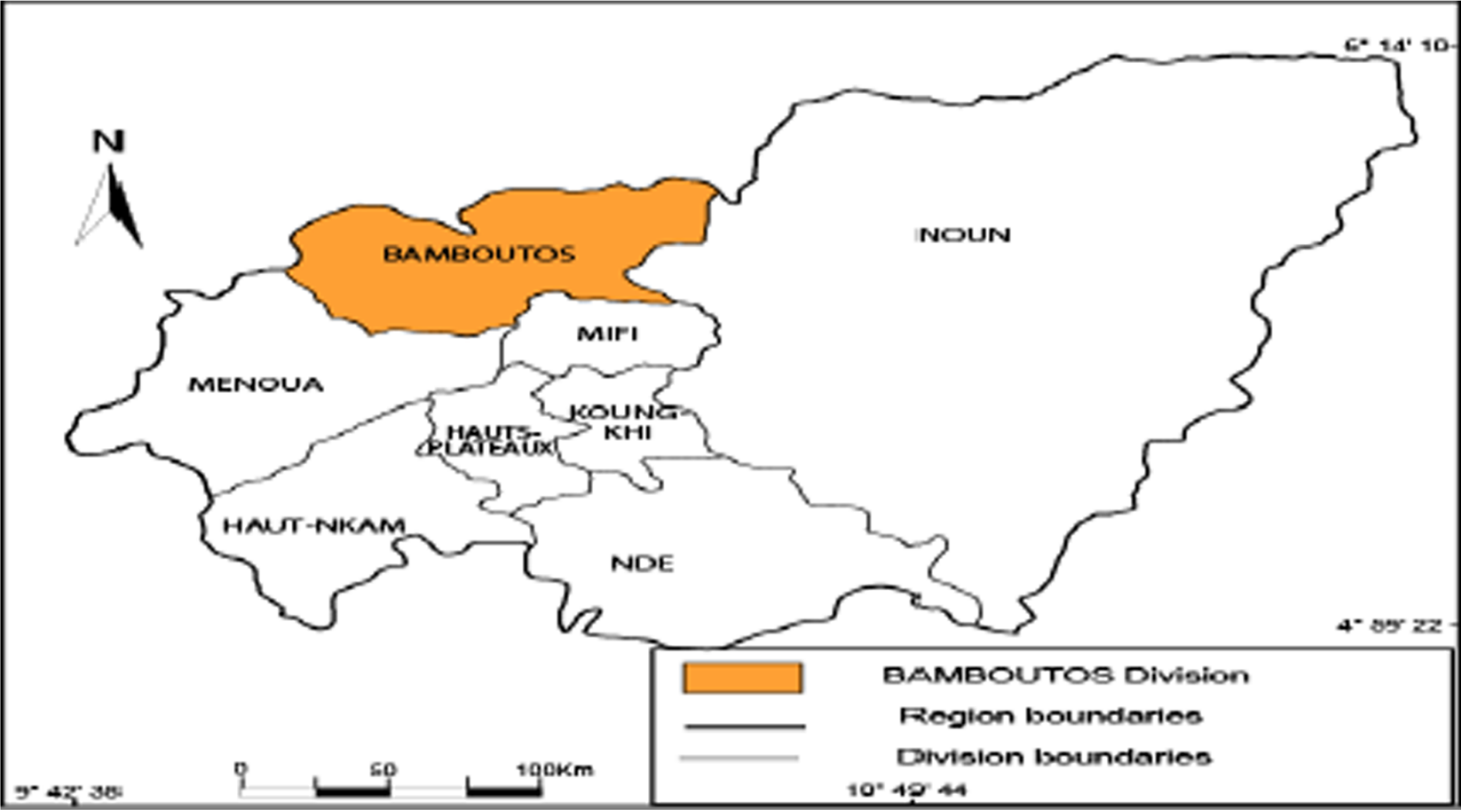
Map showing location of Bamboutos Division within West Region, Cameroon.

**Fig. 4:**
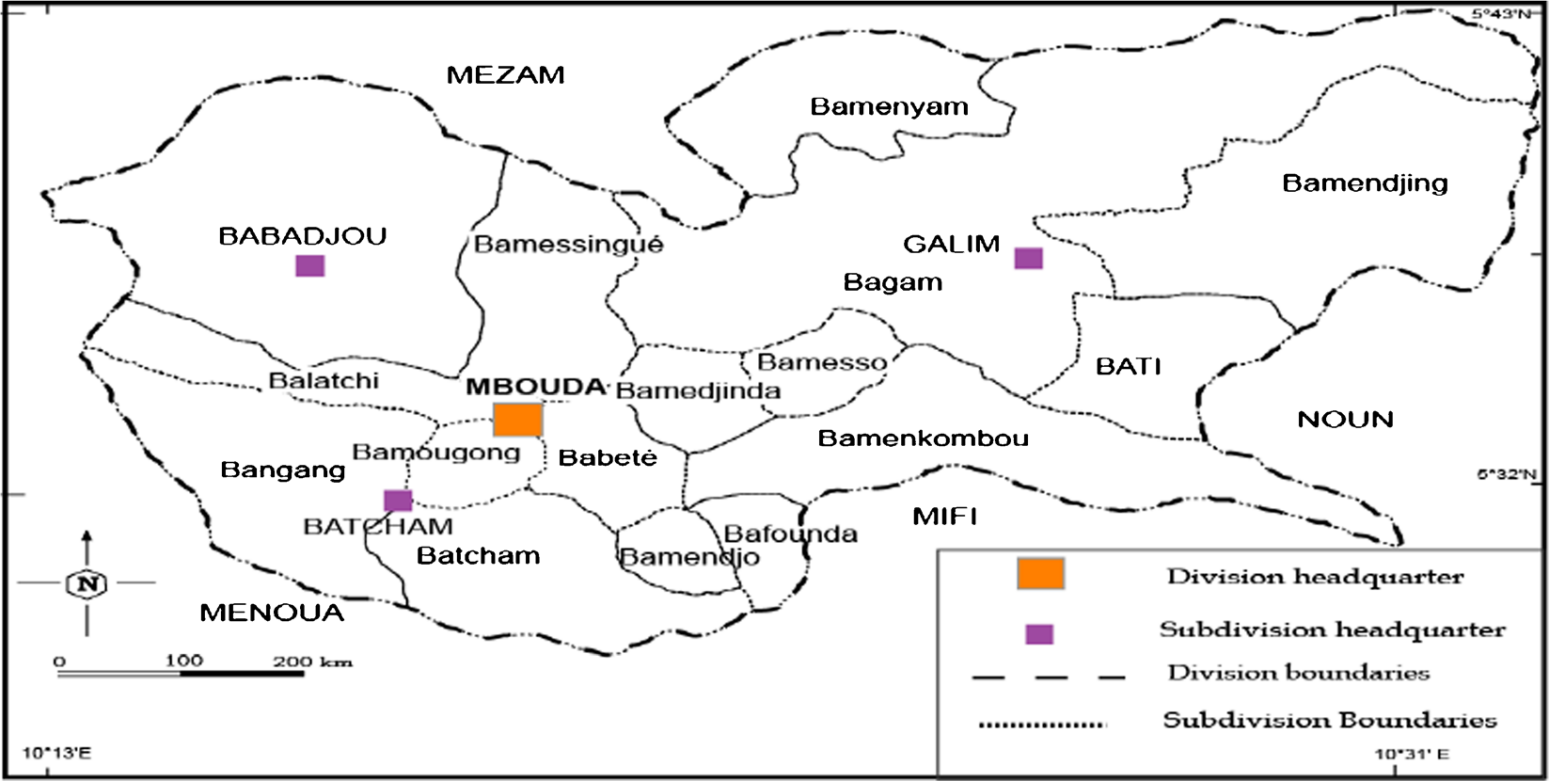
Map of Bamboutos Division within West Region of Cameroon.

The study was conducted in the Bamboutos Division that lies between 5° and 6° north latitude, and 9° and 11° east longitude. It is located 400 km west of the capital Yaoundé and is inhabited by four ethnic groups (Mboda’a, Mbofung, Megaka’a, and Mbotone). It shares borders with the Mezam Division in the North; Mifi and Menoua the South; Menoua and Manyu in the West; and Noun in the East (see Figs. 1, 2, 3, and 4). It covers an area of 1,173 km^2^ and is located in the Western Highlands of Cameroon.

The average temperature of the area is 26.8°C with a mean annual rainfall of 1,700 mm. According to MINEF (1999), Bamboutos Division has one dry season from mid–November to mid–March alternating with one rainy season that extends over the rest of the year. The highest monthly rainfall can be noted for July and August. The 2005 census data reveal that the zone had a total population of 393,410 people, representing 1.96% of the population of Cameroon (RGPH 2005). Inhabitants are mostly indigenous, but immigrants from neighboring countries and localities are also found. The majority of the population is animist, but there are also Christians and Muslims. The major vegetation in Bamboutos is alpine savannah with a cold tropical climate. The population is mainly Bamiléké and essentially consists of women, youngsters of school age, and old men, as there is a permanent rural exodus in search of jobs. The main livelihood is subsistence agriculture with small trade as sources of income.

### Selection of Respondents

Ethnobotanical data were collected between January and November 2009 from 70 respondents who were purposively selected with the help of traditional political leaders and some traditional healers in the Division and based on the availability of respondents. Rural areas were selected for this study because they are remote with poor infrastructure. Isolated communities are known to be marginalized in terms of access to health services; they also suffer high levels of poverty because they often lack appropriate means of income generation (Millar and Haverkort 2006). These factors force people to use traditional medicine for health care and often keep the indigenous knowledge associated with traditional medicine intact.

For the interviews, 70 respondents were selected using purposive sampling method (Martin 1995), of which 68 were males and 2 were females. Selected respondents were well–known in the community due to their long practice in providing services related to traditional health care. The age of the respondents ranged between 40 and 95 years (29 were between 40 and 50 years old, 18 were between 51 and 60, and 23 were over 60 years old). Forty were traditional healers; the rest were elders who had gained knowledge on medicinal uses of plants from their parents and relatives who used the plants historically.

### Ethnobotanical Data Collection

Ethnobotanical data were collected through visiting the respondents to document the indigenous knowledge regarding medicinal plants used in reproductive health, gender, socio–cultural aspects, and where the plants are harvested. Informal and formal conversations, discussions, and semi–structured interviews as well as field visits were conducted (Cotton 1996; Martin 1995). A series of individual interviews were carried out to gather information regarding name, age, sex, level of education, occupation, and ethnicity of respondents. Moreover, respondents were asked about local names of medicinal plants used, type of management (wild/cultivated), availability (common, sporadic, rare) in the area, whether their occurrence is threatened or not, reproductive health conditions treated, plant part(s) used, whether combinations of plants are used to treat any particular ailment or if any single plant is used to treat multiple ailments, condition of plant part used (fresh/dried), other ingredients or additives (if any), preparation methods, routes of remedy administration, noticeable adverse effects of remedies, taboos/beliefs related to collection and use of plants, source of knowledge, and method of indigenous knowledge transfer. Semi–structured questionnaires were also used to collect data on habit (tree, shrub, climber, herb), current habitat, and traditional conservation practices (if any) of the reported medicinal plants.

All semi–structured interviews were followed by independent walks in the field, which gave an opportunity for more discussions with respondents and practical identification of traditionally used medicinal plants in their natural environment. This method was combined with participant observation through which reliable information was obtained on the methods used during collection and preparation of specific remedial parts (Cotton 1996; Martin 1995). Interviews and discussions were conducted in local languages.

For each reported plant species, a specimen was collected, pressed, dried, and identified at the Cameroon National Herbarium Yaoundé (YA). The voucher specimen was kept at Dschang University Herbarium. Field observations were also used to record habit and habitat of each medicinal plant with the assistance of respondents interviewed.

Interviewees were chosen without distinction of gender. To guarantee respectful cooperation with respondents, we carefully explained the nature of our research and only conducted interviews after obtaining prior informed oral consent according to the Code of Ethics of the International Society of Ethnobiology (ISE 2006).

### Data Analysis

Descriptive statistics were applied to represent and list the number and percentage of species, genera, and families of medicinal plants used; their growth forms or habit, and parts used; modes of remedy preparation and routes of administration; and abundance of the species in the area and management status as described by Lulekal et al. (2008).

Based on the information obtained from respondents, reproductive ailments reported were grouped into a total of 27 categories (Table **1**, Electronic Supplementary Material, ESM). To estimate use variability of these medicinal plants and to determine which plants could be particularly interesting in the search for bioactive compounds, informant consensus factor (ICF) was calculated as follows (Heinrich et al. 1998): *ICF= N*_*ur*_*– N*_*t*_*/N*_*ur*_*– 1*, where *N*_*ur*_ is the number of use reports in each category and *N*_*t*_ is the number of species used. This factor range is from 0 to 1 and indicates the agreement among respondents on the species to be used in the treatment within an illness category. A high value (close to 1) indicates that relatively few species are used by a large proportion of the population, while a low value indicates that the respondents disagree on the species to be used for the specific treatment (Heinrich et al. 1998). A high value also indicates that the reported plant species are most frequently utilized for this illness as reported in the study by a larger number of respondents (Heinrich et al. 1998).

The family use value (FUV), which represents the relationship between the total number of plant species within a given family and the sum use values for all the species identified from the field was calculated according to Hoffman and Gallaher (2007) as follows: *FUV* = ∑ *UV*_*s*_*/N*_*s*_, where *UV*_s_ represents the use values for all species within a given plant family and N_s_ represents the total number of species within a given family.

## Results

### Diversity of Reported Medicinal Plants

The study conducted in Bamboutos Division recorded 70 medicinal plant species (Table 2, ESM) used in treating reproductive health ailments. The species belonged to 64 genera and 37 families. The Asteraceae family was represented by the highest number of species (8 species; 11.4%), followed by Euphorbiaceae (7; 10%), and Acanthaceae, Bignoniaceae, and Fabaceae (4 species each; 5.7%). Eleven of the reported families, i.e., Amaranthaceae, Amaryllidaceae, Lamiaceae, Malvaceae, Moraceae, Piperaceae, Poaceae, Rubiaceae, Solanaceae, Verbenaceae, and Vitaceae, were represented by two species each. Each of the remaining 21 families had single–species representation. Thus, 43.2% of families were represented by more than one medicinal plant species. Of all plants listed, 45.7% were herbs, 30% were shrubs, 15.7% were trees, and 8.5% were climbers.

The most commonly mentioned plant species were *Acanthus montanus* cited by 7.6% of the respondents, *Vernonia inulaefolia* (5.9%), *Cissus quadrangularis* (5.0%), and *Cyphostemma adenaucole* (4.6%), while the least–mentioned include *Aphelandra squarrosa, Calopogonium mucunoïdes, Clerodendron splendens, Polygonium nepalense*, and *Psychotria viridis* each cited by 0.1% of the respondents. Some 82.8% (58 species) of plant species mentioned were used to treat more than one reproductive health condition, whereas 17.1% (12 species) were used to treat only one ailment.

All listed plant species were mainly obtained from gardens (26.9%), grasslands (48.7%), homesteads (13.0%), and forests (9.5%); whereas, the lowest number was obtained from road sides (1.7%) (Table 3, ESM).

### Diseases Related to Reproductive Ailments

Twenty–seven (27) disease categories related to reproductive ailments were reported in Bamboutos Division (Table **1**, ESM). Among them, the venereal disease group was the one that mentioned the highest number of medicinal plants (36 species), followed by female (29 species) and male infertility (21 species); painful menstruation (dysmenorrhoea) (17 species); yellowish discharge from the vagina (leucorrhea) (17 species); vaginal cleansing (16 species); ovarian and uterus cysts (7 species); viral diseases (7 species); oligospermia (6 species); genital disease caused by *Gonococcus* bacterium (gonorrhea), irregular menstruation, inflammation of the vagina, and post–partum hemorrhage (4 species each); delivery problems, male impotence, prostate inflammation, and stimulating lactation (3 species each); abdominal pain (2 species); absence of menstruation (amenorrhoea) (2 species); cleansing a womb after giving birth (2 species); extraction of dead fetuses (2 species); fibroids (2 species); inflammation of the uterus (2 species); post–partum pain (2 species); and preventing abortion (2 species). Each of the remaining disease categories only had single–species prescriptions.

### Parts of Plants Used, Preparation Methods, and Dosage

According to interview results, leaves were the most commonly used plant part, accounting for 47.3% of total reported medicinal plant uses, followed by bark (22.3%), whole plants (19.7%), stems (5.2%), tubers 2.6%, and inflorescences and fruits (1.3%). The majority of remedies were processed as decoctions (66.2%, by boiling in water or in raphia wine), followed by crushing (25.6%, crushing fresh plant parts in water or raphia wine), maceration (6.7% in water or in raphia wine), and concoction (1.3%, with red *Elaeis guineensis* oil). The majority of remedies (75.7%) in the study area was prepared from fresh medicinal plant parts, whereas 24.3% was prepared either from dry or fresh parts. Water or raphia wine, and ingredients such as limestone, palm oil, *Citrus limon*, and *Garcinia kola* were often used in the preparation of remedies. These additives were claimed to improve flavor and taste.

Most medicinal plant preparations involved the use of single plant parts (72.8%), while mixtures of different plants or plant parts (27.1%) were encountered less in the study (Table 2, ESM). Lack of consistency regarding the amount of medicine to be used was observed among respondents during the inquiry. Most medicinal plants prescribed and given to patients are applied without any standardized doses. However, approximate dosages were reported to be determined based on age and sex of the patient and on the severity of the condition being treated.

### Route of Administration

Both internal and external applications were reported by the respondents in the treatment of reproductive health ailments in our study. Oral application (69 preparations, 98.5%) was the best–represented and most commonly used route with only one alternative administration (topical, 1.4%).

### Threats to Medicinal Plants and Conservation Practices in the Study Area

Forty–two medicinal plants (60%) were obtained from the wild, 21 (30%) were cultivated in homegardens, and 7 (10%) were either grown in homegardens or harvested from the wild. Of the total reported, 37.1% were commonly encountered, while 35.7% were considered to be presently safe, 20% were rarely encountered, and the rest (7.1%) only occur moderately or are encountered only occasionally (Table 2, ESM). In addition to the observed poor effort of cultivating medicinal plants in homegardens, it was reported that most medicinal plants are under threat due to an ever–increasing, anthropogenic pressure on the natural habitats of the medicinal plants in the study area. Respondents ranked agricultural expansion as the most serious threat to medicinal plants, followed by firewood collection, soil erosion, urbanization, over–harvesting of known medicinal species, and collecting plants for construction.

### Respondents’ Knowledge Transfer and Age

Out of the 70 key respondents, 68 were men and 2 were women. Twenty–nine were aged 40 to 50 years of age, 18 were between 51 and 60, and 23 were older than 60. In terms of education level, 47 respondents had attended basic school, whereas 23 had never attended any school. In relation to how knowledge on medicinal plants was acquired, 52 of respondents had acquired their knowledge either from their friends, parents, and/or grandparents, while 18 of them had acquired their knowledge either by themselves or from dreams. It was also found that there is maximum secrecy in passing the knowledge within the family circle. None of the respondents possessed any written documents on traditional medicine in general or on their knowledge.

### Plant Family Use Value

The plant family use value, which is applied in ethnobotany to calculate the value of a given plant taxon, helps in rating plant families for overall evaluation of member plant species in hypothesis testing, statistical validation, and comparative analysis (Hoffman and Gallaher 2007). From the results presented in Table 4 (ESM), it is clear that Vitaceae, which was represented by two plant species, was reported as the most useful family utilized in ethnomedicine in the study area, followed by Asphodelaceae, Acanthaceae, Amaryllidaceae, Euphorbiaceae, and Fabaceae, in that order.

### Informant Consensus Factor (ICF)

Plants were clustered into 27 different groups based on the use citations by respondents (Table **1**, ESM) in order to calculate the ICF. In our study, ICF values ranged from 0.5 for extraction of dead fetuses to 1.00 for acute mastitis and fat extraction around the uterus, with an average value of 0.91.

## Discussion

Indigenous people from different localities have their own specific knowledge of plant use, management, and conservation (Cotton 1996). Medicinal plants are very important for people and animals. It has been suggested that their use is one of the most significant ways in which humans directly reap the benefits provided by biodiversity (Bannister 2006; Fransworth and Soejarta 1991). During our field survey, respondents reported ethnomedicinal data on 70 species of plants distributed across 37 families and 64 genera as having properties against 27 ailments related to the reproductive health system. Among the latter families, Asteraceae was represented by eight species followed by Euphorbiaceae, which had seven species. The fact that Asteraceae is the family with the higher number of plant species in our ranking is in line with results of some studies carried out in other localities of Cameroon (Adjanohoun et al. 1996; Focho et al. 2009; Noumi 2010; Simbo 2010). These plant families are among the ones mostly seen in Cameroon (Dibong et al. 2011; Focho et al. 2009; Noumi et al. 2011). Moreover, the Asteraceae and Euphorbiaceae families were documented as dominant families in the treatment of reproductive ailments in South Western Cameroon (Focho et al. 2009). Moreover, the wide utilization of species from these families might relate to the presence of effective bioactive secondary metabolites that work against reproductive health–related infections (Cowan 1999; Gazzaneo et al. 2005; Kamatenesi-Mugisha and Oryem-Origa 2007; Kothale et al. 2011; Néné Bi et al. 2009). For example, studies have reported that the Asteraceae family is rich in monoterpenes, sesquiterpenes, sesquiterpene lactones, diterpenes, triterpenes, polyacetylenes, benzofuranes, and phenyl–propanes that help to treat various diseases (Alvarenga et al. 2001). In Egypt, Ahamed et al. (2013) found that the Euphorbiaceae family is rich in sterols, flavonoids, diterpenoids, and triterpenes that enhance and maintain body immunity. Singh et al. (2005) found that most plants used in the management of AIDS–related opportunistic infections contain flavonoids, a class of chemical compounds known to possess anti–oxidant properties that prevent free radical generation and tissue damage associated with the onset of AIDS.

Most medicinal plants used in the area were herbs. This finding is in line with results from other studies in Cameroon (Adjanohoun et al. 1996; Jiofack et al. 2010; Simbo 2010), Uganda (Kamatenesi–Mugisha et al. 2007), Ivory Coast (Djah and Danho 2011), Nigeria (Agize et al. 2013), Ethiopia (Megersa et al. 2013), and Democratic Republic of Congo (Kasali et al. 2014). This could relate to the fact that herbs are usually more readily available than shrubs and trees that are often harvested from forest patches that are distant from residential areas. It could also be due to the fact that our respondents live in shrubby savannas and grasslands where herbs abound (Simbo 2010). Our observation agrees with the general pattern of dominance of herbaceous species seen in most medicinal plant inventories in Cameroon (Focho et al. 2009; Jiofack et al. 2010), and in other African countries like Ethiopia (Agize et al. 2013; Giday et al. 2003), Uganda (Kamatenesi–Mugisha et al. 2007), and Democratic Republic of Congo (Kasali et al. 2014). Moreover, Giday et al. (2003) reported that Zay people in Ethiopia derive their medicine from herbs partly because of the fact that forests have been degraded, whereas it usually takes much more time and effort to harvest material from medicinal trees. It is true that herbs can grow everywhere (roadside, homegarden, farmland, and in wild habitats) and are common in the study area compared with other perennial life forms such as shrubs, trees, and climbers. On the other hand, the present survey is in conflict with the findings of Diame (2010), which was conducted on plants used for reproductive health at Bia Biosphere reserve in the Western region of Ghana. This survey is also in conflict with the findings of the study by Yineger and Yewhalaw (2007), which was carried out on traditional medicinal plant knowledge and used by local healers in Sekoru District, Ethiopia. It was the local healers who evidenced that trees and shrubs were the most frequently used growth forms for remedy preparation.

The majority of medicinal plants in the study area was obtained from the wild. This is in line with findings of Kamatenesi–Mugisha and Oryem–Origa (2005) in Uganda and Yineger et al. (2007) in Ethiopia, both of whom found that traditional practitioners usually collected medicinal plants from the wild. It thus appears that respondents have not yet started cultivating the majority of plant species they are using as medicine. However, the investigation showed that many of the wild habitats in the study are subjected to anthropogenic influences and consequently decreasing in size due to an ever–increasing population pressure resulting in the loss of many medicinal species sheltering in the wild.

Leaves were the most–used plant part in the preparation of drugs in the study area. This result is compared favorably to findings by Kamatenesi–Mugisha et al. (2007) and Diame (2010), who reported the widespread use of leaf remedies by the populations in Western Region Uganda and the Western Region Ghana for some gynecological morbidity and reproductive health ailments, respectively. Similar findings were also reported by Kamatenesi-Mugisha and Oryem-Origa (2007), who noted the extensive use of leaf remedies for inducing labor during childbirth in Western Uganda. The present survey is in conflict with the findings of Semenya et al. (2013), who reported that roots and bark were the most commonly harvested plant parts used to treat reproductive ailments in the Limpopo province of South Africa. It was reported that collection of roots, bark, and whole plants might kill plants in harvest (Chinsembu and Hedimbi 2010; Kamatenesi et al. 2011). Utilization of leaves may not have any detrimental effect on plants compared with plant species from which roots are utilized (Ayyanar and Ignacimuthu 2005; Poffenberger et al. 1992). Furthermore, leaves of plants have been reported to accumulate inulins, tannins, and other alkaloids more than other parts (Okoegwale and Omefezi 2001). This may be responsible for their various medicinal properties, hence explaining their wide use.

According to our results, the majority of herbal remedies in the research area were prepared from fresh materials. Other ethnobotanical inventories (Bussmann and Glenn 2010; Yineger et al. 2007) have also indicated the wide use of fresh plant material for remedy preparation probably because of the higher efficacy than when using dried plant materials. This is because some important chemicals may change in nature or even disappear upon drying.

The dominant use of medicinal plant decoctions for various ailments associated with the reproductive health system might be related to their proven effectiveness over many years of trial and indigenous knowledge accumulated on the efficacy of such preparations. On the other hand, the frequency of this method of preparation by a majority of respondents may be due to the fact that boiling the ingredients will kill some unwanted microbes that are present on the plant material used (Souad et al. 2010; Ugulu et al. 2009). Also, heat facilitates extraction of active compounds from the plant part that is an ingredient in the remedy (Souad et al. 2010). Decoction also preserves the prepared medicine longer (Muthu et al. 2006). This is in line with findings of Ugulu et al. (2009) in Izmir Province (Turkey) and Kamatenesi et al. (2011) in Northern Uganda, both of whom found that traditional practitioners in these two localities also prefer using a decoction in preparation of traditional herbal medicines.

Respondents in the study area prepare drugs for reproductive ailments, either from single plant or plant parts, or by mixing several of them. However, most remedies reported from the study area were claimed to be prepared from a single plant or plant part. Similar findings were also reported from Bolivia (Bhattarai et al. 2010) and from Mali (Togola et al. 2005). Our results deviated from those reported by Amenu (2007), who reported that 78% of traditional medicine preparations by people of Chelya Wereda (northern Ethiopia) were drawn from mixtures of different plants or plant parts. This could be attributed to the supposed additive, interactive, or synergetic effects of these mixtures (Bussmann and Sharon 2006; Igoli et al. 2002). Respondents used different additives such as limestone, *Citrus limon*, *Garcinia kola*, or palm oil in order to increase taste, flavor, and general acceptability of certain orally administered drugs. This means that since traditional medicines could have sour or bitter tastes, in most cases the additives would reduce such tastes and may even improve the efficacy of the medicine (Etana 2010).

The majority of medicines in the area were administered orally. Similar findings were reported by other researchers (Bhattarai et al. 2010; Kamatenesi et al. 2011). The choice of oral administration over possible alternatives may be related to the use of some solvents (water, palm oil, or *Raphia hookeri* wine) that are commonly believed to serve as a good vehicle to transport the remedies’ active principles. Furthermore, the lack of consistency regarding the exact amount of medicines to be used was observed among respondents during interviews. It was also reported elsewhere that the lack of precise and consistent uniform dosage is a major drawback of traditional plant use (Evans–Anfom 1986; Sofowora 1982).

As already indicated, most medicinal plants in the study area are collected for their leaves. This practice helps to reduce the pressure on these species compared to what would happen if bark or roots were collected. However, there were several medicinal plants in the study area from which bark was collected. As a result, over–collection may have a negative impact on the latter medicinal species. This was observed in the case of *Bridelia scleroneura*, *Gardenia ternifolia*, and *Vitellaria paradoxa*. Respondents reported that it is difficult to find these species specimens easily; whereas, they are increasingly rare due to over–utilization for medicinal purposes.

Respondents consistently claimed that agricultural activities are becoming the most threatening factor to medicinal plants, confirming what was reported at Bia Biosphere reserve in Western Ghana (Diame 2010) and in the Northwest Region of Cameroon (Simbo 2010). In this respect, plant species with multiple uses were said to be most affected as also witnessed during the research.

Efforts to protect medicinal plants in the study area were minimal. However, traditional belief systems may help conservation of medicinal plants, as reported by Amenu (2007) and Lulekal et al. (2008) in Ethiopia on some ethnobotanical surveys. Keeping knowledge of medicinal plants secret can also contribute to their conservation. It was reported that, if medicinal plants were to be used by more people, the threat could even increase (Mesfin et al. 2009).

Most respondents interviewed in this study were men (68 out of 70). This can be explained by the fact that men were more available in homesteads during interviews. In addition, it was not easy to locate women at home when interviews were conducted. This result is similar to those found in Mali (Togola et al. 2005); Northwest Cameroon (Simbo 2010); in villages around Kimboza forest reserve in Morogoro, Tanzania (Amri and Kisangau 2012); and with the Yoruba ethnic group of Nigeria (Kudngaongarm 2011). Our findings could also be attributed to power imbalances that favor men over women (Oyelakin 2009; Voeks 2007).

Elderly people (those older than 50 years of age) were the group most involved in traditional medicine practice. This finding is in agreement with those of Togola et al. (2005), who observed that only a few healers in their study group were younger than 40.

Intererestingly, 52 respondents had acquired their knowledge on medicinal plants from their friends, parents, and grandparents, while 18 claimed they had acquired their knowledge by themselves and/or through dreams. This supports findings in the Western Region of Ghana (Diame 2010) and in Northwestern Patagonia (Eyssartier et al. 2008). It was pointed out that in China, traditional medicinal knowledge and practices are passed orally from generation to generation (Pei 2005).

Forty–seven respondents out of 70 had completed their primary school, whereas 23 had not attended any formal schooling at all. This high illiteracy level may explain why knowledge of medicinal properties and uses of plants are not written down (Diame 2010).

Our Family Use Value shows that Vitaceae, which was represented by only two species, was reported as the most useful ethnomedicinal family in the study area followed by Asphodelaceae, Acanthaceae, Amaryllidaceae, Euphorbiaceae, and Fabaceae, some being represented by more than a single plant species. Similar results were obtained in Kenya (Gakuubi and Wanzala 2012).

*Acanthus montanus, Dyschoriste perrottettii, Eremomastax speciosa, Crinum jagus, Ageratum conyzoides, Laggera alata, Vernonia ambigua, Spathodea campanulata, Senna alata*, and *Cissus quadrangularis* were among the most–frequently utilized species. Some of these more–cited plants are also known to treat reproductive health afflictions in other parts of Cameroon (Focho et al. 2009; Noumi and Eloumou 2011), Nigeria (Soladoye et al. 2014), and Ivory Coast (Djah and Danho 2011). This may be proof of their efficacy and bioavailability. Some of the listed plants in this work have also been reported earlier in some parts of Cameroon to be useful in the management of other ailments. For instance, a decoction of *Aloe vera* leaves is used in Cameroon’s Southwest Region to treat malaria, wound, dermatitis, and poisoning (Jiofack et al. 2008). However, the diversity observed in the usage of these plants can be explained by the ecological variations observed in the different regions. This ecological variation may also influence their chemical composition, and therefore give consonance of different uses (Jiofack et al. 2010). Furthermore, if the same plant is used for the same or similar diseases in different parts of Cameroon or in different countries or continents, it may also indicate a good effect of the treatment.

Some of the survey plants have been shown to have biological activity. For example, *Aloe* spp. are known to have oestrogenic activity (Telefo et al. 2002), while *Thespesia populnea* was found to have antisteroidogenic activity in mouse ovaries (Kavimani et al. 1999). Other authors found anti–fertility effects of *Dioscorea bulbifera* (Wu et al. 2005). The analgesic activity of aqueous extracts of *Stereospermum kunthianum* stem bark was also shown (Ching et al. 2009). *Spathodea campanulata* has been reported for its immunostimulation and anti–HIV activity on mice and rats (Niyonzima et al. 1999). Some of the plant species mentioned in the present study have also been investigated for their antimicrobial activities. For example, *Ageratum conyzoides, Senna alata*, and *Solanum torvum* were found to be antimicrobial (Awal et al. 2004; Chah et al. 2000). Furthermore, it was reported that isoflavonoids isolated from *S. torvum* were found to be very active against viruses (Arthan et al. 2002).

Given that these plants either have identical uses elsewhere or their biological activities have been documented, it has been suggested that a similar use of a medicinal plant by different people from different areas can be considered to be a good and reliable indicator of the validity of the species’ curative properties (Lans 2007; Zerbo et al. 2007).

In our study, medicinal plant species claimed to cure venereal diseases had the highest ICF, followed by those used to treat female and male infertility, dysmenorrhoea, and leucorrhoea and for vaginal cleaning; the lowest ICF value was recorded for medicinal plants only used for dead fetal extraction. However, this value was equal to 0.5, which would typically result from a plant use to treat rare diseases (Tabuti et al. 2012), suggesting that our survey addressed medicinal plant species commonly used for treating reproductive health ailments in the study area. The high level of consensus among respondents about the uses of medicinal plants for the treatment of reproductive health care diseases prevalent in the study area suggests that the ethnomedicinal uses of these plants are currently still in widespread practice in the study area.

## Conclusion

Traditional medicine remains the most affordable and most easily accessible source of treatment in the primary health system of rural populations worldwide.

The present study documents 70 medicinal plants and their uses. The majority of the species were harvested from the wild with herbs representing the highest proportion. The majority of species was harvested for their leaves, a harvest strategy that causes minimal damage to the plants compared to those that are harvested for their roots or bark. High numbers of medicinal plants reported to be used for treating reproductive health ailments are being threatened by different human activities while conservation efforts are minimal in the area. Agricultural expansion was reportedly the most serious threat to medicinal plants in the study area. To save medicinal plants from further loss, local populations should be educated on sustainable methods of harvesting. Cultivation and domestication of at least the most rare and most highly used plant species are needed.

The preservation of these plant species is the gateway towards developing efficacious remedies for continuing to treat the mentioned diseases.

Moreover, reported plant species can serve as a basis for formal analysis of active compounds and validation of results.

## Electronic supplementary material

Below is the link to the electronic supplementary material.

Table 1 (DOCX 29 kb)

Table 2 (DOCX 50 kb)

Table 3 (DOCX 26 kb)

Table 4 (DOCX 23 kb)
