# Supplementary material for: Medicinal Plants Used for Treating Reproductive Health Care Problems in Cameroon, Central Africa1
Source: Econ Bot. 2016 May 17;70:145–59. doi: 10.1007/s12231-016-9344-0 (PMC4927590; doi:10.1007/s12231-016-9344-0)
Supplement: Supplementary file 1 — (DOCX 29 kb) [file 12231_2016_9344_MOESM1_ESM.docx]

<TT>Table 1. Informant consensus factor (ICF) values of use categories of plants claimed as having medicinal values by respondents.

| **<TH>Use Category** | **Plant Species** | **N_TAXA_** | **Number of Use Citation** | **IFC**  **Value** |
| --- | --- | --- | --- | --- |
| <TB>Abdominal pains | *Piper capense* (14), *Solanum torvum* (9) | 2 | 14 | 0.92 |
| Amenorrhoea | *Achyranthes aspera* (1) *Bridelia scleroneura* (12) | 2 | 13 | 0.92 |
| Fat extraction around uterus | *Calopogonium mucunoides* (2) | 1 | 2 | 1.00 |
| Dysmenorrhoea | *Dyschoriste perrottettii* (12), *Eremomastax speciosa* (23), *Aloe barbadense* (25), *Achyranthes aspera* (1), *Rauvolfia vomitoria* (3), *Crassocephalum mannii* (8), *Laggera alata* (12), *Vernonia inulaefolia* (35), *Spathodea campanulata* (11), *Bryophyllum pinnatum* (8), *Bridelia scleroneura* (12), *Croton macrostachyus* (10), *Entada abyssinica* (10), *Satureja robusta*(2), *Thespesia populnea* (7), *Lippia multiflora* (3), *Cyphostemma adenaucole* (27) | 17 | 209 | 0.92 |
| Cleansing womb after giving birth | *Ceratopteris cornuta* (2), *Solanum torvum* (9) | 2 | 11 | 0.90 |
| Extraction of the dead fetuses | *Caucalis melanantha* (1), *Ceratopteris cornuta* (2) | 2 | 3 | 0.50 |
| Female infertility | *Spathodea campanulata* (11), *Elaephorbia drupifera* (20)*, Euphorbia lateriflora* (20)*, Aloe barbadense* (25)*, Cissus quadrangularis* (30) *Cyphostemma adenaucole* (27), *Acanthus montanus* (45), *Eremomastax speciosa* (23), *Furcraea foetida* (5), *Cyathula cylindrica* (1), *Crinum jagus* (13), *Rauvolfia vomitoria* (3), *Polyscias fulva* (7), *Vernonia ambigua* (15), *Vernonia cf inulaefolia* (35), *Kigelia africana* (9), *Sanseviera liberica* (3), *Bridelia scleroneura* (12), *Croton macrostachyus* (10), *Piper capense* (14), *Ricinus communis* (3), *Entada abyssinica* (10), *Clerodendron splendens* (1), *Satureja robusta* (2), *Thespesia populnea* (7), *Polygonium nepalense* (1), *Gardenia ternifolia* (2), *Solanum torvum* (9), *Lippia multiflora* (3) | 29 | 366 | 0.92 |
| Fibroids | *Elaephorbia drupifera* (20), *Ficus exasperata* (8) | 2 | 28 | 0.96 |
| Gonorrhoea | *Acanthus montanus* (45), *Eremomastax speciosa* (23), *Melinis minutiflora* (2), *Physalis micrantha* (2) | 4 | 72 | 0.96 |
| Helping delivery of newborn | *Crassocephalum mannii* (8), *Markhamia tomentosa* (5), *Hibiscus noldea* (6) | 3 | 19 | 0.89 |
| Irregular menstruation or menstrual regulation | *Acanthus montanus* (45), *Eremomastax speciosa* (23), ), *Entada abyssinica* (10), *Satureja robusta*(2) | 4 | 80 | 0.96 |
| Inflammation of the uterus | *Vernonia ambigua* (15), *Senna alata* (22), | 2 | 37 | 0.97 |
| Inflammation of the vagina | *Ageratum conyzoides* (21), *Kigelia Africana* (9), *Entada abyssinica* (10), *Thespesia populnea* (7) | 4 | 47 | 0.93 |
| Leucorrhea | *Spathodea campanulata* (11), *Acanthus* montanus (45),Dyschoriste *perrottettii* (12), *Eremomastax speciosa* (23), *Rauvolfia vomitoria* (3), *Crassocephalum mannii* (8), *Laggera alata* (12), *Vernonia inulaefolia* (35), *Impatiens burtonii* (1), *Basella alba* (7), *Kigelia africana*(11), *Bryophyllum pinnatum* (8), *Gladiolus undulates* (1), *Entada abyssinica* (10), *Thespesia populnea* (7), *Pittosporum mannii* (3), *Lippia multiflora*(3) | 17 | 200 | 0.92 |
| Male infertility | *Spathodea campanulata* (11), *Euphorbia lateriflora* (20)*, Aloe barbadensis* (25)*, Cissus quadrangularis* (30), *Acanthus* montanus (45), *Eremomastax speciosa* (23), *Furcraea foetida* (5), *Cyathula cylindrical* (1), *Crinum natans* (13), *Rauvolfia vomitoria* (3), *Polyscias fulva* (7), *Vernonia ambigua* (15), *Vernonia inulaefolia* (35), *Kigelia africana* (9), *Sanseviera liberica* (3), *Croton macrostachyus* (10), *Entada abyssinica* (10), *Satureja robusta* (2), *Thespesia populnea* (7), *Gardenia ternifolia* (2), *Solanum torvum* (9) | 21 | 285 | 0.93 |
| Male impotence | *Vernonia* sp. (2), *Clerodendron splendens* (1), *Thespesia populnea* (7) | 3 | 10 | 0.78 |
| Painful breast or acute mastitis | *Piper umbellatum* (11) | 1 | 11 | 1.00 |
| Post partum Hemorrhage | *Achyranthes aspera* (1), *Musa sapientum* (9), *Setaria megaphylla* (4), *Vitex doniana* (19) | 4 | 33 | 0.91 |
| Post partum pain | *Eremomastax speciosa* (23), *Piper capense* (14)  **Table 1 : Continued** | 2 | 37 | 0.97 |
| Prostate inflammation | *Kigelia africana* (9), *Euphorbia lateriflora* (20), *Gardenia ternifolia* (2) | 3 | 31 | 0.93 |
| Stimulating lactation | *Euphorbia lateriflora* (20), *Satureja robusta* (2), *Vitellaria paradoxa* (4) | 3 | 26 | 0.92 |
| Oligospermia | *Rauvolfia vomitoria* (3), *Sonchus angustissimus* (3), *Sonchus oleraceus* (3), *Impatiens burtonii* (1), *Basella alba* (7), *Euphorbia lateriflora* (20) | 6 | 37 | 0.89 |
| Ovarian and uterus cysts | *Caucalis melanantha* (1), *Elaephorbia drupifera* (20)*, Ficus exasperata* (8), *Ceratopteris cornuta* (2), *Gardenia ternifolia* (2), *Cyphostemma adenaucole* (27), *Vernonia ambigua* (15) | 7 | 75 | 0.92 |
| Preventing abortion | *Zehneria scabra* (6), *Hibiscus noldeae* (6) | 2 | 12 | 0.91 |
| Vaginal cleaning | *Spathodea campanulata* (11), *Elaephorbia drupifera* (20)*, Euphorbia lateriflora* (20)*, Aloe barbadense* (25)*, Cissus quadrangularis* (30), *Cyphostemma adenaucole* (27), *Acanthus montanus* (45), *Rauvolfia vomitoria* (3), *Polyscias fulva* (7), *Ageratum conyzoides* (21), *Vernonia ambigua* (15), *Vernonia inulaefolia* (35), *Kigelia africana* (9), *Bryophyllum pinnatum* (8), *Senna alata* (22), *Gardenia ternifolia* (2) | 16 | 300 | 0.95 |
| Venereal diseases | *Spathodea campanulata* (11), *Euphorbia lateriflora* (20)*, Cissus quadrangularis* (30), *Acanthus montanus* (45), *Aphelandra squarrosa*(1), *Furcraea foetida* (5), *Rauvolfia vomitoria* (3), *Polyscias fulva* (7), *Laggera alata* (12), *Sonchus angustissimus* (3), *Sonchus oleraceus* (3), *Vernonia ambigua* (15), *Vernonia inulaefolia* (35), *Vernonia sp* (2), *Kigelia africana* (9), *Combretum smeathmannii* (1), *Ipomoea batatas* (3), *Scleria pterota* (1), *Dioscorea dumetorum* (1), *Sanseviera liberica* (3), *Gladiolus undulates* (1), *Croton macrostachyus* (10), *Macaranga sp* (1), *Phyllanthus amarus* (9), *Ricinus communis* (3), *Entada abyssinica* (10), *Mimosa invisa* (5), *Senna alata* (22), *Clerodendron splendens* (1), *Satureja robusta* (2), *Thespesia populnea* (7), *Ficus sur* (4), *Gardenia ternifolia* (2), *Psychotria viridis* (1), *Smilax kraussiana* (2) , *Stereospermum accuminatissimum* (4) | 36 | 294 | 0.88 |
| Viral diseases | *Rauvolfia vomitoria* (3), *Vernonia inulaefolia* (35), *Stereospermum accuminatissimum* (4), *Euphorbia lateriflora* (20), *Phyllanthus amarus* (9), *Gardenia ternifolia* (2), *Smilax kraussiana* (2) | 7 | 75 | 0.92 |
| Total | | 202 | 2327 | 0.91 |

<TFN>Numbers between brackets indicate the number of citations of that plant by respondents (traditional healers and elders) against particular ailments related to the reproductive system.

N_TAXA_= number of taxa used to treat that particular category as mentioned by respondents.
