# Supplementary material for: Medicinal Plants Used for Treating Reproductive Health Care Problems in Cameroon, Central Africa1
Source: Econ Bot. 2016 May 17;70:145–59. doi: 10.1007/s12231-016-9344-0 (PMC4927590; doi:10.1007/s12231-016-9344-0)
Supplement: Supplementary file 2 — (DOCX 50 kb) [file 12231_2016_9344_MOESM2_ESM.docx]

<TT>Table 2. Medicinal plants used for the treatment of reproductive health care problems in Bamboutos Division, West Region, Cameroon.

| **<TH>Species and Family** | **Local name (language)** | **Habit** | **Parts Used** | **Voucher Number** | **Ailments Treated** | **Mode of Preparation and Administration** | **Number of Citations** |
| --- | --- | --- | --- | --- | --- | --- | --- |
| <TB>*Acanthus montanus* (Nees) T. Anders  Acanthaceae | *Megigoum* (*Ngomba’a*)  *Mounamenang*  (Ngiembon)  *Megigor* (*Megaka*) | Herb | Whole plant | 9067/SRFCam | Irregular Menstruation;  Male and female infertility;  Gonorrhea;  Leucorrhoea;  Venereal diseases;  Vaginal cleansing | Decoction in raphia wine of whole plant with stem bark of *Spathodea campanulata*, leaves of *Elaephorbia drupifera, Euphorbia lateriflora, Aloe barbadensis, Cissus quadrangularis* and *Cyphostemma adenaucole* taken orally | 45 |
| *Aphelandra squarrosa* Nees  Acanthaceae | — | Shrub | Leaves | Non applicable | Venereal diseases | Decoction in raphia wine with limestone taken orally | 1 |
| *Dyschoriste perrottettii* (Nees) Kuntze  Acanthaceae | *Mekonbi* (*Ngomba’a*) | Herb | Whole plant | 19413/SRFCam | Leucorrhoea;  Dysmenorrhoea | Maceration in water taken orally | 12 |
| *Eremomastax speciosa* (Hochst) Cufod.  Acanthaceae | *Pankuzem* (*Ngomba’a*)  *Panzemok* (*Ngiembon*)  *Piezeumok* (*Megaka*)  *Kouokmegar* (*Megaka*) | Shrub | Leaves | 16371 SRFCam | Post–partum pain;  Male and female infertility;  Leucorrhoea;  Dysmenorrhoea;  Gonorrhea;  Irregular menstruation | Maceration in water taken orally | 23 |
| *Furcraea foetida* (L.) Haw.  Agavaceae | *Melan* (*Ngomba’a*) | Herb | Leaves | 25568/SRFCam | Male and female infertility;  Venereal diseases | Decoction in raphia wine taken orally | 5 |
| *Achyranthes aspera* L.  Amaranthaceae | *Koudjo’o* (*Ngomba’a*) | Herb | Leaves | 61065/HNC | Post–partum hemorrhage;  Amenorrhoea;  Dysmenorrhoea | Crushed extract, taken orally | 1 |
| *Cyathula cylindrica* Moq.  Amaranthaceae | — | Herb | Whole plant | 35028/HNC | Male and female infertility | Crushed extract, taken orally | 1 |
| *Crinum jagus* (J. Thomps) Dandy  Amaryllidaceae | *Melan bène* (*Ngomba’a*)  *Melan bang* (*Ngiembon*) | Shrub | Whole plant | 32871/HNC | Female infertility | Decoction in raphia wine taken orally | 13 |
| *Crinum natans* Baker  Amaryllidaceae | *Melan paguè* (*Ngomba’a*)  *Melan pazuè* (*Ngiembon*) | Herb | Whole plant | 18263/SRFCam | Male infertility | Decoction in raphia wine taken orally | 13 |
| *Caucalis melanantha* Benth. & Hook.f. syn. *Agrocharis melanantha* Hochst.  Hiern  Apiaceae | _ | Herb | Leaves | 22007/SRFCam | Extraction of death fetus;  Ovarian cysts | Crushed extract, taken orally | 1 |
| *Rauvolfia vomitoria* Afzel.  Apocynaceae | *Nepemo* (*Ngiembon*) | Shrub | Bark | 2143/SRFK | Male and female infertility;  Viral and venereal diseases;  Leucorrhoea;  Oligospermia;  Dysmenorrhoea;  Vaginal cleansing | Decoction in raphia wine or water taken orally | 3 |
| *Polyscias fulva* (Hiern) Harms  Araliaceae | *Kikiokdiore* (*Ngomba’a*) | Tree | Bark | 2990/SRFCam | Male and female infertility;  Venereal diseases;  Vaginal cleansing | Decoction in raphia wine with leaves of *Elaephorbia drupifera, Euphorbia lateriflora, Cissus quadrangularis* and *Cyphostemma adenaucole* taken orally | 7 |
| *Aloe barbadense* Mill. syn. *Aloe vera* (L.) Burm.f.  Asphodelaceae | *Melan* (*Ngomba’a*)  *Vougnang* (Megaka) | Herb | Leaves | Non applicable | Male and female infertility;  Male impotence;  Dysmenorrhoea;  Vaginal cleansing | Decoction in raphia wine with *Euphorbia lateriflora, Cissus quadrangularis* and *Acanthus montanus* taken orally | 25 |
| *Ageratum conyzoides* Linn.  Asteraceae | *Tsomamou* (*Ngomba’a*)  *Tchouamou* (*Ngiembon*)  *Nekouadar* (*Ngomba’a* & *Ngiembon*)  *Soumomo*(*Megaka*) | Herb | Whole plant | 6575 SRFCam | Vaginal cleansing;  Inflammation of the vagina | Crushed extract, taken orally | 21 |
| *Crassocephalum mannii* (Hook. f.)Milne–Redh.  Asteraceae | *Neponlou* (*Ngomba’a*)  *Poupou* (*Ngomba’a*) | Shrub | Leaves | 7623/SRFCam | Helping delivery;  Leucorrhoea;  Dysmenorrhoea | Crushed extract, taken orally | 8 |
| *Laggera alata* (D.Don) Sch. Bip ex Oliv.  Asteraceae | *Depack–kenan* (*Ngomba’a*) | Shrub | Leaves | 6567/SRFCam | Venereal diseases;  Leucorrhoea;  Dysmenorrhoea | Decoction in raphia wine taken orally | 12 |
| *Sonchus angustissimus* Hook.f.  Asteraceae | *Kuku–movou* (*Ngomba’a*) | Herb | Leaves | 16944/SRFCam | Male impotence;  Oligospermia;  Venereal diseases | Crushed extract with pulverized seeds of *Garcinia kola* taken orally | 3 |
| *Sonchus oleraceus* Linn.  Asteraceae | *Kuku* (*Megaka* & *Ngiembon*) | Herb | Leaves | 53051/HNC | Male impotence;  Oligospermia;  Venereal diseases | Crushed extract with pulverized seeds of *Garcinia kola* taken orally | 3 |
| *Vernonia ambigua* Kotschy & Peyr  Asteraceae | *Negi–gouan* (*Ngomba’a*) | Shrub | Leaves | 47156/HNC | Vaginal cleansing;  Male and female infertility;  Venereal diseases;  Inflammation of the uterus | Crushed extract, taken orally | 15 |
| *Vernonia cf inulaefolia* Steud. ex Walp.  Asteraceae | *Vougnang* (*Megaka*)  *Megoutsop* (*Megaka*) | Herb | Tuber | 37876/HNC | Vaginal cleansing;  Male and female infertility;  Viral and venereal diseases;  Dysmenorrhoea;  Leucorrhoea | Decoction or maceration with *Citrus lemon* taken orally | 35 |
| *Vernonia sp*  Asteraceae | *Depak–kenan* (*Ngomba’a*) | Shrub | Leaves | Non applicable | Venereal diseases;  Male impotence | Crushed extract, taken orally | 2 |
| *Impatiens burtonii* Hook. F  Balsaminaceae | — | Herb | Whole plant | 27760/SRFCam | Oligospermia;  Leucorrhoea | Crushed extract, taken orally | 1 |
| *Basella alba* Linn.  Basellaceae | *Ndote* (*Megaka*, *Ngomba’a* & *Ngombalé*) | Climber | Whole plant | 6467/SRFCam | Oligospermia;  Leucorrhoea | Crushed extract, taken orally | 7 |
| *Kigelia africana* (Lam.) Benth  Bignoniaceae | *Sacktare* (*Ngombalé* & *Ngomba’a*)  *Vinvi* (*Megaka*)  Kebi (Megaka) | Tree | Fruit  Bark | 23220/SRFCam | Vaginal cleansing;  Male and female infertility;  Inflammation of the Vagina;  Venereal diseases;  Prostate inflammation | Decoction in raphia wine with bark of *Stereopermum accuminatissimum* and *Spathodea campanulata* taken orally | 9 |
| *Markhamia tomentosa* (Benth.) K. Schum. ex. Engl  Bignoniaceae | *Warè* (*Ngomba’a*)  *Wate–kufo* (*Ngiembon*) | Tree | Bark, leaves | 1974/SRFCam | Helping delivery | Decoction or crushed extract in raphia wine of leaves with leaves of *Hibiscus noldea* and *Bryophyllum pinnatum* taken orally | 5 |
| *Spathodea campanulata* P. Beauv  Bignoniaceae | *Foukfoukgue* (*Ngomba’a*) | Tree | Bark | 29470/HNC | Vaginal cleansing;  Dysmenorrhoea;  Leucorrhoea;  Venereal diseases;  Male and female infertility | Decoction in raphia wine with whole plant of *Acanthus montanus, Cissus quadriangularis*, stem of *Cyphostemma adenaucole* and *Kigelia africana* taken orally | 11 |
| *Stereospermum accuminatissimum* K. Schum.  Bignoniaceae | *Wate–fè* (*Ngomba’a &* *Ngombalé*)  *Ware–fè* (*Ngomba’a*)  *Siton* (*Megaka*) | Tree | Bark | 45705/HNC | Viral and venereal diseases | Decoction in raphia wine with whole plant of Acanthus montanus, *Cissus quadriangularis,* stem of *Cyphostemma adenaucole, Kigelia africana* and *Spathodea campanulata* taken orally | 4 |
| *Combretum smeathmannii* G.Don., syn. *C. macronatum* Schumach & Thonn.  Combretaceae | — | Climber | Leaves | 43685/HNC | Venereal diseases | Decoction in raphia wine taken orally | 1 |
| *Ipomoea batatas* (L.) Lam.  Convolvulaceae | *Kopgoua* (*Ngomba’a*)  *Voukope* (*Megaka*) | Herb | Leaves  Stem | 9588/SRFCam | Venereal diseases | Crushed extract, taken orally | 3 |
| *Bryophyllum pinnatum* (Lam.) Oken  Crassulaceae | *Nezouk* (*Ngomba’a*)  *Lezouk* (*Ngiembon*) | Herb | Leaves | 33394/HNC | Vaginal cleansing;  Leucorrhoea;  Dysmenorrhoea | Crushed extract, is taken orally | 8 |
| *Zehneria scabra* (L.F). Sonder  Cucurbitaceae | *Liepliep* (*Ngomba’a*)  *Laplap* (*Ngomba’a*)  *Leliep* (*Ngiembon*) | Herb | Leaves | 23723/SRFCam | Preventing abortion or spurious labour | Maceration taken orally | 6 |
| *Scleria pterota* K. Presl ex. C.B. Clarke. syn. *Scleria melaleuca* Rchb. ex Schltdl.& Cham.  Cyperaceae | *Monipefo* (*Megaka* & *Ngomba’a*)  *Cesoue* (*Megaka*) | Herb | Whole plant | 35211/HNC | Venereal diseases | Decoction in raphia wine taken orally | 1 |
| *Dioscorea dumetorum* (Kunth) Pax  Dioscoreaceae | *Neliock* (*Ngomba’a*)  *Zouck* (*Megaka*) | Climber | Leaves | 24431/SRFCam | Venereal diseases | Decoction in water or raphia wine taken orally | 1 |
| *Sanseviera liberica* Hort. ex. Gérôme & Labroy  Dracaenaceae | *Melannagoue* (*Ngomba’a*)  *Lack* (*Ngiembon*) | Herb | Leaves | 43510/HNC | Male and female infertility;  Venereal diseases | Decoction in raphia wine taken orally | 3 |
| *Gladiolus undulates* L.  Iridaceae | — | Herb | Tuber | Non applicable | Venereal diseases;  Leucorrhoea | Decoction in raphia wine taken orally | 1 |
| *Bridelia scleroneura* Müll. Arg.  Euphorbiaceae | *Mezene* (*Ngomba’a*) | Tree | Bark | 3268/SRFK | Female infertility;  Dysmenorrhoea;  Amenorrhoea | Decoction in raphia wine with whole plant of *Acanthus montanus, Cissus quadriangularis,* stem of *Cyphostemma adenaucole, Kigelia africana* and *Spathodea campanulata* taken orally | 12 |
| *Croton macrostachyus* Hochst. ex. Delile  Euphorbiaceae | *Tsam* (*Megaka* & *Ngomba’a*)  *Tsa’a* (*Megaka* & *Ngomba’a*) | Tree | Bark | 40501/HNC | Venereal diseases;  Male and female infertility;  Dysmenorrhoea | Decoction in raphia wine with whole plant of *Acanthus montanus, Elaephorbia drupifera, Euphorbia lateriflora* and *Piper capense* taken orally | 10 |
| *Elaephorbia drupifera* syn. *Euphorbia drupifera* Thonn.  Euphorbiaceae | *Machoir* (*Ngomba’a*) | Shrub | Leaves | 39169/HNC | Vaginal cleansing;  Female infertility;  Fibroids;  Ovarian cysts | Decoction in raphia wine with whole plant of *Acanthus montanus, Croton macrostachys, Euphorbia lateriflora* and *Piper capense* taken orally | 20 |
| *Euphorbia lateriflora* Schumach.  Euphorbiaceae | *Nepimou* (*Ngomba’a*) | Shrub | Stem | 37987/HNC | Vaginal cleansing;  Male and female infertility;  Viral and venereal diseases;  Prostate inflammation;  Promoting lactation in women after giving birth;  Oligospermia | Decoction in raphia wine with whole plant of *Acanthus montanus, Elaephorbia drupifera, Croton macrostachys* and *Piper capense*, taken orally | 20 |
| *Macaranga sp*  Euphorbiaceae | *Deupfo* (*Ngiembon*)  *Deutpou* (*Ngiembon*) | Shrub | Leaves, Bark | Non applicable | Venereal diseases | Decoction in raphia wine taken orally | 1 |
| *Phyllanthus amarus* Schumach.  Euphorbiaceae | — | Herb | Whole plant | 17780/SRFCam | Viral and venereal diseases | Decoction in water or raphia wine taken orally | 9 |
| *Ricinus communis* Linn.  Euphorbiaceae | — | Shrub | Leaves | 4405/SRFCam | Venereal diseases;  Female infertility | Decoction in water with limestone taken orally | 3 |
| *Calopogonium mucunoides* Desv.  Fabaceae | — | Herb | Leaves | 17456/SRFCam | Fat extraction around uterus | Decoction in water taken orally | 2 |
| *Entada abyssinica* Steud. ex A. Rich.  Fabaceae | *Cessan* (*Megaka*)  *Lou* (*Ngiembon*) | Tree | Bark | 26967/SRFCam | Male and female infertility;  Venereal diseases;  Dysmenorrhoea;  Leucorrhoea;  Inflammation of the vagina;  Menstrual regulation | Decoction in raphia wine taken orally | 10 |
| *Mimosa invisa* Martius ex Colla syn. *M. diplotricha* Sauvalle  Fabaceae | *Samedji* (*Ngomba’a*) | Herb | Leaves | 45022/HNC | Venereal diseases | Decoction in raphia wine taken orally | 5 |
| *Senna alata* (Linn.) Roxb  Fabaceae | *Foupan* (*Ngomba’a* & *Ngiembon*) | Shrub | Leaves | 42258/HNC | Vaginal cleansing;  Inflammation of the uterus;  Venereal diseases | Decoction in water taken orally | 22 |
| *Clerodendron splendens* G.Don.  Lamiaceae | — | Herb | Leaves | 50234/HNC | Venereal diseases;  Female infertility;  Male impotence | Decoction in water taken orally | 1 |
| *Satureja robusta* (Hook. f) Brenan. syn. *Clinopodium robustum* (Hook.f.) Ryding  Lamiaceae | *Poumouo* (*Megaka*) | Herb | Whole plant | 54416/HNC | Promoting lactation in women after birth;  Male and female infertility;  Venereal diseases;  Dysmenorrhoea;  Menstrual regulation | Decoction or crushed extract, in water or raphia wine taken orally | 2 |
| *Hibiscus noldeae* Bak.f.  Malvaceae | *Samedji* (*Ngomba’a*) | Herb | Leaves | 17546/SRFCam | Helping delivery of newborn;  Preventing abortion | Crushed extract in water, taken orally | 6 |
| *Thespesia populnea* (L.) Sol. ex. Corrêa  Malvaceae | *Kepfou* (*Megaka*, *Ngombalé* & *Ngomba’a*)  *Tekue* (*Ngiembon*) | Shrub | Leaves | 36533/HNC | Venereal diseases;  Dysmenorrhoea;  Male impotence;  Male and female infertility;  Inflammation of the vagina;  Leucorrhoea | Crushed extract, taken orally | 7 |
| *Ficus exasperata* Vahl  Moraceae | *Kokguème* (*Ngomba’a*) | Shrub | Leaves  Bark | 5123/SRFK | Ovarian and uterus cysts;  Fibroids | Decoction or crushed extract, taken orally | 8 |
| *Ficus sur* Forssk  Moraceae | *Gack* (*Ngomba’a*) | Tree | Bark | 37426/HNC | Venereal diseases | Decoction in raphia wine taken orally | 4 |
| *Musa sapientum* Linn.  Musaceae | *Kedilack* (*Ngomba’a* & *Ngombalé*)  *Kadilack* (*Ngiembon*) | Herb | Inflorescences | Not found | Postpartum hemorrhage | Decoction in raphia wine taken orally | 9 |
| *Ceratopteris cornuta* (P.Beauv) Lepr.  Parkeriaceae | — | Herb | Leaves | 28496/SRFCam | Extraction of the dead fetus;  Cleansing womb after childbirth;  Ovarian cysts | Maceration in water taken orally | 2 |
| *Piper capense* L.f.  Piperaceae | *Bepote–goo* (*Ngomba’a*) | Shrub | Leaves | 44108/HNC | Female infertility  Postpartum abdominal Pain | Decoction in raphia wine with whole plant of *Acanthus montanus, Elaephorbia drupifera, Croton macrostachys, Euphorbia lateriflora* and *Piper capense*, taken orally | 14 |
| *Piper umbellatum* Linn.  Piperaceae | *Bepote* (*Megaka*, *Ngomba’a*, *Ngiembon* & *Ngombalé*) | Shrub | Leaves | 19813/ SRFCam | Acute mastitis or Painful breast | Crushed leaves applied topically on the breast | 11 |
| *Pittosporum mannii* Hook.F syn. *P. viridiflorum* Sims  Pittosporaceae | — | Shrub | Bark | 23851/SRFCam | Leucorrhoea | Decoction in raphia wine taken orally | 3 |
| *Setaria megaphylla* (Steud) Durand & Schunz  Poaceae | *Samedji* (*Ngomba’a*)  *Soussongkane* (*Ngomba’a*) | Herb | Whole plant | 17727/SRFCam | Postpartum hemorrhage | Decoction in raphia wine with bark of *Vitex doniana*, taken orally | 4 |
| *Melinis minutiflora* P.Beauv.  Poaceae | *Voutguiè* (*Megaka*)  *Nguetpuiék* (*Megaka*) | Herb | Whole plant | 35516/HNC | Gonorrhea | Decoction in water or raphia wine taken orally | 2 |
| *Polygonium nepalense* Meisn. syn. *Persicaria nepalensis* (Meisn.) Miyabe  Polygonaceae | — | Herb | Whole plant | 16818/SRFCam | Female infertility | Decoction in water taken orally | 1 |
| *Gardenia ternifolia* Schumach & Thonn.  Rubiaceae | *Metoucbouor* (*Ngomba’a*)  *Metobouo* (*Megaka*) | Shrub | Leaves, Bark | 40092/HNC | Male and female infertility;  Viral and venereal diseases;  Vaginal cleansing;  Ovarian and uterus cysts;  Prostate inflammation | Decoction in water or raphia wine taken orally | 2 |
| *Psychotria viridis* Ruiz & Pav.  Rubiaceae | — | Shrub | Bark | Non applicable | Venereal diseases | Decoction in water or raphia wine taken orally | 1 |
| *Vitellaria paradoxa* C. F. Gaertn  Sapotaceae | *Saloboué* (*Megaka*)  *Sack–kouket* (*Megaka*) | Tree | Bark | 12856/HNC | Promoting lactation in women after giving birth | Decoction in raphia wine taken orally | 4 |
| *Smilax kraussiana* Meisn. syn. *Smilax anceps* Willd.  Smilacaceae | *Bounk* (*Ngiembon*)  *Bii* (*Megaka*) | Climber | Leaves | 22614/SRFCam | Venereal diseases;  Viral diseases | Decoction in raphia wine taken orally | 2 |
| *Physalis micrantha* Link. syn. *P. lagascae* Roem. & Schult.  Solanaceae | *Djidjimoching* (*Ngomba’a*)  *Bateum* (*Megaka*)  *Deudeupmochin* (*Megaka*) | Herb | Whole plant | 8982/SRFCam | Gonorrhea | Decoction or crushed extract, taken orally | 2 |
| *Solanum torvum* Sw. syn. *S. rudepannum* Dunal  Solanaceae | *Tchiè* (*Ngomba’a*) | Shrub | Leaves | 14615/ SRFCam | Male and female infertility;  Abdominal pains;  Cleansing womb after childbirth | Fried leaves with palm oil taken orally | 9 |
| *Lippia multiflora* Moldenke  Verbenaceae | *Bounkmong* (*Megaka*) | Herb | Leaves | 18856/SRFCam | Female infertility;  Leucorrhoea;  Dysmenorrhoea; | Decoction in raphia wine taken orally | 3 |
| *Vitex doniana* Sweet.  Verbenaceae | *Vounetane* (*Ngomba’a*) | Tree | Bark | 49905/HNC | Postpartum hemorrhage | Decoction in raphia wine with bark of *Setaria megaphylla*, taken orally | 15 |
| *Cissus quadrangularis* Linn.  Vitaceae | *Djicmetsi* (*Ngiembon* & *Ngomba’a*) | Climber | Stem | 7739/SRFCam | Male and female infertility;  Venereal Diseases;  Vaginal cleansing | Decoction in raphia wine of whole plant with stem bark of *Spathodea campanulata,* leaves of *Elaephorbia drupifera, Euphorbia lateriflora, Aloe barbadensis, Acanthus montanus* and *Cyphostemma adenaucole* taken orally | 30 |
| *Cyphostemma adenaucole* (Stendel. ex–A. Rich.) Wild & R.B. Drumm.  Vitaceae | *Pioktè* (*Ngomba’a*) | Climber | Stem | 7743/SRFCam | Female infertility;  Dysmenorrhoea;  Vaginal cleansing;  Ovarian and uterus cyst | Decoction in raphia wine of whole plant with stem bark of *Spathodea campanulata,* leaves of *Elaephorbia drupifera, Euphorbia lateriflora, Aloe barbadensis, Cissus quadrangularis* and *Acanthus montanus*, taken orally | 27 |

<TFN>— = Vernacular name not found; *Ngomba’a, Ngiembon, Ngombalé* and *Megaka* in bracket belong to ethnolinguistic groups.

**Table 1 (end)**
