# Supplementary material for: Medicinal Plants Used for Treating Reproductive Health Care Problems in Cameroon, Central Africa1
Source: Econ Bot. 2016 May 17;70:145–59. doi: 10.1007/s12231-016-9344-0 (PMC4927590; doi:10.1007/s12231-016-9344-0)
Supplement: Supplementary file 3 — (DOCX 26 kb) [file 12231_2016_9344_MOESM3_ESM.docx]

<TT>Table 3. Medicinal plant status and habitat in Bamboutos Division.

| **<TH>Species and Family** | **Status of Plant** | **Abundance Status or Availability** | **Habitat** |
| --- | --- | --- | --- |
| <TB>*Acanthus montanus*  Acanthaceae | Wild | Common | Grassland, under big trees and, in the forest |
| *Aphelandra squarrosa* Acanthaceae | Cultivated | Presently safe | Gardens |
| *Dyschoriste perrottettii*  Acanthaceae | Wild | Common | Grassland and  road sides |
| *Eremomastax speciosa* Acanthaceae | Semi–Cultivated | Presently safe | Gardens |
| *Furcraea foetida* Agavaceae | Cultivated | Presently safe | Gardens and  homesteads |
| *Achyranthes aspera*  Amaranthaceae | Wild | Common | Grassland |
| *Cyathula cylindrica*  Amaranthaceae | Wild | Moderately or occasionally encountered | Forest and grassland |
| *Crinum jagus*  Amarylidaceae | Cultivated | Presently safe | Gardens and  homesteads |
| *Crinum natans*  Amaryllidaceae | Cultivated | Presently safe | Gardens and homesteads |
| *Caucalis melanantha* syn. *Agrocharis melanantha*  Apiaceae | Wild | Moderately or occasionally encountered | Grassland |
| *Rauvolfia vomitoria*  Apocynaceae | Semi–Cultivated | Presently safe | Gardens and homesteads |
| *Polyscias fulva* Araliaceae | Semi–Cultivated | Presently safe | Forest and grassland |
| *Aloe barbadense*  syn. *Aloe vera* Asphodelaceae | Cultivated | Presently safe | Gardens |
| *Ageratum conyzoides* Asteraceae | Wild | Common | Grassland and road sides |
| *Crassocephalum mannii*  Asteraceae | Cultivated | Presently safe | Gardens and homesteads |
| *Laggera alata*  Asteraceae | Wild | Common | Grassland and gardens |
| *Sonchus angustissimus*  Asteraceae | Wild | Common | Grassland |
| *Sonchus oleraceus* Asteraceae | Wild | Common | Grassland |
| *Vernonia ambigua*  Asteraceae | Cultivated | Presently safe | Gardens and homesteads |
| *Vernonia cf inulaefolia*  Asteraceae | Wild | Rare | Grassland |
| *Vernonia sp*  Asteraceae | Wild | common | Grassland |
| *Impatiens burtonii* Balsaminaceae | Wild | Common | Forest |
| *Basella alba* Basallaceae | Cultivated | Presently safe | Gardens and homesteads |
| *Kigelia africana*  Bignoniaceae | Cultivated | Presently safe | Forest,  Grassland and  gardens |
| *Markhamia tomentosa*  Bignoniaceae | Semi–cultivated | Presently safe | Grassland and homesteads |
| *Spathodea campanulata*  Bignoniaceae | Semi–cultivated | Presently safe | Grassland, homesteads and gardens |
| *Stereospermum accuminatissimum* Bignoniaceae | Semi–cultivated | Presently safe | Grassland, homesteads and  gardens |
| *Combretum smeathmannii*  syn. *C. macronatum* Combreatceae | Wild | Rare | Grassland |
| *Ipomoea batatas* Convolvulaceae | Cultivated | Presently safe | Gardens |
| *Bryophyllum pinnatum*  Crassulaceae | Semi–cultivated | Common | Gardens and grassland |
| *Zehneria scabra* Cucurbitaceae | Wild | Common | Under big trees and gardens edges |
| *Scleria pterota*  syn. *Scleria melaleuca* Cyperaceae | Wild | Rare | Grassland |
| *Dioscorea dumetorum*  Dioscoreaceae | Cultivated | Presently safe | Gardens |
| *Sanseviera liberica*  Dracaenaceae | Wild | Rare | Grassland and gardens edges |
| *Gladiolus undulatus*  Eridaceae | Cultivated | Presently safe | Gardens |
| *Bridelia scleroneura*  Euphorbiaceae | Wild | Rare | Grassland, Forest |
| *Croton macrostachyus*  Euphorbiaceae | Wild | Common | Grassland and in the forest |
| *Elaephorbia drupifera* syn. *Euphorbia drupifera*  Euphorbiaceae | Cultivated | Common | Gardens |
| *Euphorbia lateriflora* Euphorbiaceae | Cultivated | Presently safe | Gardens |
| *Macaranga sp*  Euphorbiaceae | Wild | Rare | Grassland and gardens edges |
| *Phyllanthus amarus* Euphorbiaceae | Wild | Common | Forest |
| *Ricinus communis* Euphorbiaceae | Wild | Common | Grassland |
| *Calopogonium mucunoides*  Fabaceae | Wild | Common | Grassland |
| *Entada abyssinica*  Fabaceae | Wild | Presently safe | Grassland and edge of gardens |
| *Senna alata*  Fabaceae | Cultivated | Presently safe | Gardens |
| *Clerodendron splendens*  Lamiaceae | Cultivated | Rare | Grassland |
| *Satureja robusta*  syn. *Clinopodium robustum*  Lamiaceae | Wild | Rare | Grassland |
| *Hibiscus noldea*  Malvaceae | Wild | Common | Grassland and homesteads |
| *Thespesia populnea*  Malvaceae | Wild | Common | Grassland and homesteads |
| *Mimosa invisa*  syn. *M. diplotricha*  Fabaceae | Wild | Common | Grassland |
| *Ficus experata* Moraceae | Wild | Common | Grassland |
| *Ficus sur*  Moraceae | Cultivated | Presently safe | Grassland |
| *Musa sapientum*  Musaceae | Cultivated | Presently safe | Gardens |
| *Ceratopteris cornuta*  Parkeriaceae | Cultivated | Rare | Grassland |
| *Piper capense* Piperaceae | Wild | Common | Forest, Grassland |
| *Piper umbellatum*  Piperaceae | Wild | Common | In the forest and grassland |
| *Pittosporum mannii* syn. *P. viridiflorum*  Pittosporaceae | Wild | Moderately or occasionally encountered | Grassland |
| *Setaria megaphylla* Poaceae | Wild | Moderately or occasionally encountered | Gardens, in the forest and grassland |
| *Melinis minutiflora*  Poaceae | Wild | Moderately or occasionally encountered | Grassland |
| *Polygonium nepalense* syn. *Persicaria nepalensis* Polygonaceae | Wild | Common | Grassland |
| *Gardenia ternifolia*  Rubiaceae | Wild | Rare | Grassland, |
| *Psychotria viridis* Rubiaceae | Wild | Rare | Grassland |
| *Vitellaria paradoxa* Sapotaceae | Wild | Rare | Homesteads and grassland |
| *Smilax kraussiana* syn. *Smilax anceps*  Smilacaceae | Wild | Common | Grassland |
| *Physalis micrantha* syn. *P. lagascae*  Solanaceae | Wild | Rare | Gardens, homesteads and grassland |
| *Solanum torvum*  syn. *S. rudepannum*  Solanaceae | Cultivated | Presently safe | Gardens, homesteads and grassland |
| *Lippia multiflora*  Verbenaceae | Wild | Rare | Grassland |
| *Vitex doniana* Verbenaceae | Wild | Common | Grassland |
| *Cissus quadrangularis*  Vitaceae | Cultivated | Presently safe | Gardens and grassland |
| *Cyphostemma adenaucole*  Vitaceae | Wild | Common | Gardens and grassland |
