# Supplementary material for: Medicinal Plants Used for Treating Reproductive Health Care Problems in Cameroon, Central Africa1
Source: Econ Bot. 2016 May 17;70:145–59. doi: 10.1007/s12231-016-9344-0 (PMC4927590; doi:10.1007/s12231-016-9344-0)
Supplement: Supplementary file 4 — (DOCX 23 kb) [file 12231_2016_9344_MOESM4_ESM.docx]

<TT>Table 4. Analysis of documented plant species by family use values (n = 37)

| **<TH>Family** | **Number of Species** | **% of all Species** | **Number of Respondent Use Citations** | **% use Citations** | **Family Use Value** |
| --- | --- | --- | --- | --- | --- |
| <TB>Acanthaceae | 4 | 5.7 | 81 | 13.71 | 0.289 |
| Agavaceae | 1 | 1.4 | 5 | 0.84 | 0.071 |
| Amaranthaceae | 2 | 2.8 | 2 | 0.33 | 0.014 |
| Amaryllidaceae | 2 | 2.8 | 26 | 4.40 | 0.186 |
| Apiaceae | 1 | 1.4 | 1 | 0.17 | 0.014 |
| Apocynaceae | 1 | 1.4 | 1 | 0.17 | 0.014 |
| Araliaceae | 1 | 1.4 | 7 | 1.18 | 0.1 |
| Asphodelaceae | 1 | 1.4 | 25 | 4.23 | 0.357 |
| Asteraceae | 8 | 11.4 | 99 | 16.75 | 0.177 |
| Balsaminaceae | 1 | 1.4 | 1 | 0.17 | 0.014 |
| Basellaceae | 1 | 1.4 | 7 | 1.18 | 0.1 |
| Bignoniaceae | 4 | 5.7 | 29 | 4.91 | 0.103 |
| Combretaceae | 1 | 1.4 | 1 | 0.17 | 0.014 |
| Convovulaceae | 1 | 1.4 | 3 | 0.51 | 0.043 |
| Crassulaceae | 1 | 1.4 | 8 | 1.35 | 0.114 |
| Cucurbitaceae | 1 | 1.4 | 6 | 1.01 | 0.085 |
| Cyperaceae | 1 | 1.4 | 1 | 0.17 | 0.014 |
| Dioscoreaceae | 1 | 1.4 | 1 | 0.17 | 0.014 |
| Dracaenaceae | 1 | 1.4 | 3 | 0.51 | 0.043 |
| Iridaceae | 1 | 1.4 | 1 | 0.17 | 0.014 |
| Euphorbiaceae | 7 | 10 | 75 | 12.69 | 0.153 |
| Fabaceae | 4 | 5.7 | 39 | 6.60 | 0.139 |
| Lamiaceae | 2 | 2.8 | 3 | 0.51 | 0.021 |
| Malvaceae | 2 | 2.8 | 13 | 2.20 | 0.093 |
| Moraceae | 2 | 2.8 | 12 | 2.03 | 0.093 |
| Musaceae | 1 | 1.4 | 9 | 1.52 | 0.128 |
| Parkeriaceae | 1 | 1.4 | 2 | 0.33 | 0.028 |
| Piperaceae | 2 | 2.8 | 25 | 4.23 | 0.179 |
| Pittosporaceae | 1 | 1.4 | 3 | 0.51 | 0.043 |
| Poaceae | 2 | 2.8 | 6 | 1.01 | 0.043 |
| Polygonaceae | 1 | 1.4 | 1 | 0.17 | 0.014 |
| Rubiaceae | 2 | 2.8 | 3 | 0.51 | 0.021 |
| Sapotaceae | 1 | 1.4 | 4 | 0.68 | 0.057 |
| Smilacaceae | 1 | 1.4 | 2 | 0.33 | 0.029 |
| Solanaceae | 2 | 2.8 | 11 | 1.86 | 0.079 |
| Verbenaceae | 2 | 2.8 | 18 | 3.05 | 0.129 |
| Vitacaeae | 2 | 2.8 | 57 | 9.64 | 0.407 |
